# Asymptomatic Preclinical Rheumatoid Arthritis-Associated Interstitial Lung Disease

**DOI:** 10.1155/2013/406927

**Published:** 2013-07-31

**Authors:** Juan Chen, YongHong Shi, XiaoPing Wang, Heqing Huang, Dana Ascherman

**Affiliations:** ^1^Rheumatology Department of the First Hospital of Xiamen University, Zhen Hai Road No. 55, Xiamen 361003, China; ^2^Pulmonary Department of the First Hospital of Xiamen University, Zhen Hai Road No. 55, Xiamen 361003, China; ^3^Radiology Department of the First Hospital of Xiamen University, Zhen Hai Road No. 55, Xiamen 361003, China; ^4^University of Miami School of Medicine, Miami, FL 33136, USA

## Abstract

*Objective*. Interstitial lung disease (ILD) is a common extra-articular manifestation of rheumatoid arthritis (RA) and a significant cause of morbidity and mortality. The objective of this study was to define high-resolution chest CT (HRCT) and pulmonary function test (PFT) abnormalities capable of identifying asymptomatic, preclinical forms of RA-ILD that may represent precursors to more severe fibrotic lung disease. *Methods*. We analyzed chest HRCTs in consecutively enrolled RA patients and subsequently classified these individuals as RA-ILD or RA-no ILD based on the presence/absence of ground glass opacification, septal thickening, reticulation, traction bronchiectasis, and/or honeycombing. Coexisting PFT abnormalities (reductions in percent predicted FEV1, FVC, TLC, and/or DLCO) were also used to further characterize occult respiratory defects. *Results*. 61% (63/103) of RA patients were classified as RA-ILD based on HRCT and PFT abnormalities, while 39% (40/103) were designated as RA-no ILD. 57/63 RA-ILD patients lacked symptoms of significant dyspnea or cough at the time of HRCT and PFT assessment. Compared with RA-no ILD, RA-ILD patients were older and had longer disease duration, higher articular disease activity, and more significant PFT abnormalities. *Conclusion*. HRCT represents an effective tool to detect occult/asymptomatic ILD that is highly prevalent in our unselected, university-based cohort of RA patients.

## 1. Introduction

Interstitial lung disease (ILD) is a common extra-articular manifestation responsible for significant morbidity and mortality among patients with rheumatoid arthritis (RA) [1]. These findings parallel the relationship between ILD and other systemic autoimmune diseases such as systemic sclerosis, inflammatory myopathy (polymyositis and dermatomyositis), Sjogren's syndrome, and undifferentiated CTD [1–3]. 

A recently published population-based study showed that the hazard ratio for development of *clinically evident* ILD in RA (compared to individuals without RA) was 8.96 [4]. Moreover, the risk of death for RA patients with ILD was 3 times higher than in RA patients without ILD, and median survival after ILD diagnosis was only 2.6 years [4]. Given the morbidity/mortality associated with progressive/advanced RA-ILD, earlier diagnosis and aggressive management are critical. 

While the majority of cases of RA-ILD occur in patients between the ages of 50 and 60 years, smoking, male gender, and longstanding RA represent additional risk factors for the development of ILD [2]. Patients with established RA-ILD most often present with chronic symptoms of dyspnea and cough. Physical examination may reveal inspiratory crackles, and pulmonary function tests (PFTs) typically demonstrate restrictive physiology marked by a reduced diffusing capacity [2, 5]. Imaging abnormalities are variable, but often demonstrate traction bronchiectasis and/or honeycombing suggestive of usual interstitial pneumonia (UIP) [1].

Based on high-resolution computed tomography (HRCT) scanning, the estimated prevalence of RA-associated ILD (RA-ILD) among patients with RA varies widely from 4 to 68%-depending on patient selection and severity of pulmonary symptoms [2]. With plain chest radiography, the frequency of ILD detection is only 5%. In a minority of cases, surgical lung biopsy may ultimately be required to establish the diagnosis and histopathological subtype [4, 6]. Because asymptomatic, preclinical ILD demonstrable by HRCT can be progressive and may be more prevalent than expected among patients having RA [7], the objective of this study was to characterize and define the frequency of HRCT and PFT abnormalities capable of identifying “subclinical” RA-ILD. 

## 2. Material and Methods

### 2.1. Study Population

Patients with RA were consecutively recruited through the Rheumatology Department at The First Hospital of Xiamen University, China, between July 1, 2012 and March 1, 2013. Enrolment into these cohorts was approved by the ethics committee of the First Hospital of Xiamen University and included permission to review all medical records. Demographics, clinical features, medication history, comorbidities, and the disease activity score calculated for 28 joints (DAS28) were carefully recorded. The swollen or tender joint count (0–28) component of the DAS28 included shoulder joints (2), elbow joints (2), wrist joints (2), metacarpophalangeal joints (10), proximal interphalangeal joint (10), and knee joints (2). HRCTs and PFTs were obtained in 100% and 80.6% of study eligible patients, respectively.

### 2.2. Inclusion Criteria

All enrolled patients were older than 18 years of age and satisfied ACR criteria for definite RA [8]. Eligible patients were further classified into categories of RA-ILD and RA-no ILD based on the following criteria:

1. RA-ILD-HRCT abnormalities consisting of ground glass opacities, septal lines, reticulation, subpleural fibrosis, traction bronchiectasis, architectural distortion, and/or honeycombing. These radiographic abnormalities could occur with/without clinical symptoms of dyspnea and cough or significant pulmonary function test (PFTs) abnormalities defined as <80% predicted values for the following parameters: forced expiratory volume in 1 second (FEV1), forced vital capacity (FVC), total lung capacity (TLC), and diffusion capacity of carbon monoxide (DLCO),
2. RA-no ILD—absence of significant HRCT or PFT abnormalities as defined earlier.

### 2.3. Exclusion Criteria

Individuals with active infection, HIV/AIDS, malignancy, or posttransplant immunosuppression were excluded from enrollment due to potential confounding effects on data analysis.

### 2.4. High-Resolution Computed Tomography

High-resolution computed tomography (HRCT) of the chest without intravenous contrast medium was performed during end inspiration with the patient in a supine position using 1-2 mm collimation at 1-2 mm intervals (Toshiba Medical Systems, Aquilion 16 (Japan)). Images were read independently by 2 blinded pulmonary and radiology specialists (YongHong Shi and XiaoPing Wang), with a focus on the previously outlined parenchymal lung abnormalities. Discrepant readings were rereviewed to determine consensus findings. Using a modification of a previously described quantitative scale [7], reviewers assigned HRCT scores according to the following criteria: 0 (normal), 1 (minimal disease (i.e., 3-4 septal lines)), 2 (mild disease (>5 septal lines, reticulations, subpleural cysts, or ground glass opacities)), 3 (moderate disease (i.e., grade 2 findings as well as traction bronchiectasis, peri-bronchovascular thickening, or tracheal retraction with one-third to two-thirds lung involvement)), or 4 (severe disease (i.e., grade 2 or 3 findings with more than two-thirds lung involvement)).

### 2.5. Pulmonary Function Test (PFTs)

Pulmonary function was assessed according to American Thoracic Society recommendations [9] and expressed as percent predicted FEV1, FVC, TLC, and DLCO. 

### 2.6. Statistical Analyses

Statistical analyses were performed using SPSS software. All descriptive data were expressed as mean values ± SD. While Student's *t*-test was used to compare normally distributed quantitative data, chi-squared testing with Yates correction was used to compare frequencies. In all analyses, a two-tailed *P* value less than 0.05 was considered statistically significant. 

## 3. Results

The clinical data of 103 consecutively enrolled RA patients (76 women and 27 men) are shown in Table 1. For the overall cohort, mean age was 49.1 ± 14.7 years (range 19–81), and the mean disease duration was 4.3 ± 5.7 years. The mean articular disease activity score (DAS28) was 4.4 ± 1.4. 

61% (63/103) of RA patients were diagnosed with RA-ILD by HRCT and PFTs, while 39% (40/103) did not meet criteria for ILD and were therefore designated as RA-no ILD. 57/63 RA-ILD patients (90% of RA-ILD subset) lacked symptoms of significant dyspnea or cough at the time of HRCT and PFTs assessment, whereas 6 patients (10% of RA-ILD subset) manifested these clinical features. Compared with patients in the RA-no ILD subgroup, RA-ILD patients were older (53.0 ± 14.8 versus 42.9 ± 12.4 years, *P* < 0.001) and had longer disease duration (5.0 ± 6.7 versus 3.1 ± 3.0 years, *P* < 0.05), greater articular disease activity (DAS 28 scores: 4.9 ± 1.2 versus 3.7 ± 1.3, *P* < 0.001), and more severe respiratory defects (lower percent predicted FVC, TLC, FEV1, and DLCO; all with *P* < 0.001) (Tables 1 and 2). As shown in Table 1, there were no statistically significant differences in the levels of RF or anti-CCP antibodies between the two groups. 

Evaluation of chest HRCT scores (Table 3) revealed that the percentage of individuals among the overall RA cohort with radiographic abnormalities was 78.4% (81/103). The main HRCT abnormality was ground glass opacification suggestive of nonspecific interstitial pneumonia (NSIP), a defect that was observed in 57.3% (59/103) of RA patients. Of note, ground glass opacification was detected far more frequently in RA-ILD relative to RA-no ILD patients (83% versus 18%, *P* < 0.001). Ten patients manifested mild ground glass opacification in single lung lobe (7/40), airspace abnormalities (1/40), or architectural distortion (2/40). They were identified as RA-no ILD based on associated comorbidities. By comparison, 0/24 age- and sex-matched healthy volunteers had HRCT evidence of ground glass opacification, septal thickening, traction bronchiectasis, or honeycombing.

In this study, all RA patients were treated with combinations of DMARDs that included methotrexate (MTX), with a dose ranging from 10 to 15 mg per week; however, only 7.8% of RA patients (8/103) used biologics or TNF-*α* blocking agents. In terms of additional environmental risk factors, the prevalence of smoking was very low in both the RA-ILD and RA-no ILD subsets (1/63 and 1/40, resp.), with only one individual in each subgroup having smoking exposure greater than 25 pack-years [10]. Overall, the lack of differences in treatment regimen and the relatively low frequency of smoking in the overall cohort collectively precluded meaningful statistical correlation of these variables with the presence/absence of ILD.

## 4. Discussion

Asymptomatic/preclinical RA-ILD, which is detectable by HRCT and pulmonary function tests, was observed in 55% of RA patients in our study. The overall prevalence of radiographically defined RA-ILD in this unselected cohort of Chinese RA patients was quite high (61%), though the frequency of *clinically evident* ILD (6%) was consistent with the existing literature [4]. Based on comparative analysis of clinical and demographic variables, older age, longstanding disease, and higher disease activity represent risk factors for the development of RA- ILD [4].

Previous studies have shown that asymptomatic/preclinical RA-ILD is relatively prevalent and often progressive, as evidenced by the analysis of Gochuico et al. showing worsening HRCT scores over a 2-year period in 12/21 individuals with subclinical ILD [7]. Further demonstrating the relatively high prevalence of subclinical RA-ILD, another study reported HRCT evidence of pulmonary disease in 50% of RA patients, with only 10% of these patients manifesting clinical symptoms of lung involvement [11]. 

In separate analyses, Kim et al. highlighted the wide range of RA-ILD prevalence reported in the literature (as low as 4% and as high as 68%) [2]. To better address this issue and the implications for disease progression/survival, he and his research group defined the HRCT characteristics suggestive of usual interstitial pneumonia (UIP) (basilar predominant reticulation, traction bronchiectasis, and honeycombing, with limited ground glass abnormality) versus NSIP (predominant bibasilar ground glass attenuation with limited to no reticulation and absence of honeycombing) [1], showing a much worse survival in those with radiographic findings indicative of UIP (hazard ratio of 2.3) [1, 4]. 

Using somewhat different criteria, Biederer et al. demonstrated that patients with RA-ILD and a predominantly ground glass pattern seen on HRCT scans (likely indicative of NSIP) had a shorter mean duration of RA than those patients with a reticular pattern (63 ± 38 months versus 133 ± 112 months, resp.; *P* < 0.01) [12]. In our study where the main HRCT abnormality was ground glass opacification resembling a NSIP pattern, the mean disease duration of 4.3 ± 5.7 years was similar to that reported by Biederer et al. but shorter than that reported by Zou et al. in a cohort of Chinese RA patients (8.25 ± 9 years) [13]. The latter discrepancy may reflect the wider range of histopathological subtypes included in the analysis of Zou et al. [13], as UIP typically develops in more longstanding disease [4, 12]. 

What remains unknown is the natural history of subclinical RA-ILD with underlying NSIP histopathology and whether this subtype represents a precursor to end-stage fibrotic lung disease. By our study, however, HRCT is a sensitive measurement to identify asymptomatic preclinical ILD that could reflect pathologic patterns more amenable to immunomodulatory therapy [1, 2, 4, 6]. Therefore, obtaining chest HRCTs early in disease (even in the absence of clinical symptoms) to select patients for more aggressive pharmacologic therapy may represent an effective strategy for halting progression to more fibrotic stages of lung disease (such as UIP) that carry a dismal prognosis.

In our analysis, pulmonary function test abnormalities served as another sensitive indicator for preclinical RA-ILD, paralleling findings of other studies where pulmonary function parameters (lower FEV1, FVC, TLC, and DLCO) in RA-ILD patients correlated with HRCT abnormalities [4]. Of note, Hamblin and Horton found that a reduced DLCO of less than 54% of the predicted value appears to be an independent predictor of ILD progression in RA [10] though the applicability of this finding to subclinical RA-ILD (where impairment of DLCO is generally less severe) remains unclear. 

Based on our data, we are unable to conclude whether smoking, MTX, or other unknown medication/environmental exposures increase the risk for development of RA-ILD. Answering these questions will require larger, longitudinal studies incorporating more RA patients. Complementary strategies involving the assessment of serum protein biomarkers should further elucidate disease pathogenesis and provide additional insight regarding the relationship between subclinical RA-ILD and end-stage, fibrotic lung disease that portends a devastating outcome. 

The prevalence of asymptomatic, subclinical RA-ILD defined by HRCT and PFT abnormalities was 55% in our cohort of RA patients. The predominant HRCT abnormality among this subset of RA-ILD patients was ground glass opacification most suggestive of NSIP. Older age, longstanding disease, and higher articular disease activity strongly correlated with the development of RA-ILD. Overall, chest HRCT represents a highly sensitive technique for detection of RA-ILD, even in the absence of clinical symptoms, making this procedure an effective screening tool for early ILD that may allow institution of more aggressive therapy geared towards the prevention of end-stage, fibrotic lung disease.

## Figures and Tables

**Table 1:** Comparison of clinical characteristics in RA-ILD and RA-no ILD.

	All	RA-ILD	RA-no ILD	*P* value
Total number	103	63	40	
Gender (F/M)	76/27	46/17	30/10	0.823
Mean age (year)	49.1 ± 14.7	53.0 ± 14.8	42.9 ± 12.4	<0.001*
Mean disease duration (years)	4.3 ± 5.7	5.0 ± 6.7	3.1 ± 3.0	0.049*
DAS28	4.4 ± 1.4	4.9 ± 1.2	3.7 ± 1.3	<0.001*
RF	402.0 ± 1268.2	555.8 ± 1599.1	159.8 ± 206.7	0.057
Anti-CCP	218.2 ± 171.7	231.8 ± 178.0	196.8 ± 161.1	0.316

*P < 0.05.

**Table 2:** Pulmonary function tests in RA-ILD and RA-no ILD.

% of predicted	RA-ILD (*n* = 47)	RA-no ILD (*n* = 36)	*P* value
FEV1	74.1 ± 14.6	88.0 ± 12.9	<0.001
FVC	74.9 ± 12.2	86.9 ± 11.3	<0.001
TLC	87.7 ± 15.7	98.4 ± 11.3	0.001
DLCO	68.1 ± 19.5	96.2 ± 17.7	<0.001

**Table 3:** Lung HRCT scores of RA-ILD and RA-no ILD.

HRCT findings	RA-ILD (*n* = 63)	RA-no ILD (*n* = 40)	*P* value
Ground glass	52	7	<0.001*
Airspace	6	1	NS
Mixed AS/GG	4	0	NS
Honeycombing	4	0	NS
Architectural distortion	20	2	NS
Traction bronchiectasis	11	0	NS

*P < 0.05.
